# Molecular Mechanisms of Plant Defense against Abiotic Stress

**DOI:** 10.3390/ijms241210339

**Published:** 2023-06-19

**Authors:** Emilia L. Apostolova

**Affiliations:** Institute of Biophysics and Biomedical Engineering, Bulgarian Academy of Sciences, Acad. G. Bonchev Str., Bl. 21, 1113 Sofia, Bulgaria; emya@bio21.bas.bg or emilia.apostolova@gmail.com

## Abstract

The climatic changes and anthropogenic factors in recent decades (global warming, drought, salinity, extreme temperature, environmental pollution) have led to an increase in the negative impact of environmental factors on plants. Abiotic stress strongly influences the important processes of plants and thus affects their growth and development. The effects of stressors on the plants depend on the intensity, frequency, and duration of stress, plant species as well as a combination of various stressors. Plants have developed different mechanisms to limit adverse environmental conditions. In the publications in this Special Issue, Molecular Mechanisms of Plant Defense against Abiotic Stress, new information on plant defense mechanisms against abiotic and biotic stress is presented. The studies help us better understand plants' protection mechanisms again global climate change.

## 1. Introduction

Plants are continually subjected to environmental stresses, which harm crop development and yield. Based on FAO (Food and Agriculture Organization) reports, 96.5% of agricultural land is affected by abiotic stress factors [1], which influence the plant grown and developed, affecting the gene expression and some important physiological, biochemical, and processes [2]. Adaptation to environmental stressors involves integrative responses to individual stresses and a new type of response [3]. Extreme environmental conditions increase the amount of reactive oxygen species (ROS) and cause oxidative stress [4]. The antioxidant enzyme systems and non-enzymatic antioxidants are critical for plant protection against oxidative damage [5]. Stressors alter the expression of different genes [6]. In the last few years, the role of hyperosmolality-gated calcium-permeable channels (OSCA) as osmosensors during plant development and stress responses has been shown. The OSCA gene family was studied in different plant species [7,8]. In this Special Issue, the study of Kaur et al. [9] identified a total of 42 OSCA genes in *Triticum aestivum* and revealed the probable function of these genes in the plant development and stress response in this plant species. In continuous climate change conditions, pests' influence increases on the plants. The immune defense system of plants and its important regulatory role in plant defense against pests are presented in this Special issue [10].

## 2. Salt Stress

Soil salinity is an environmental problem worldwide, affecting around 20% of agricultural land [11]. Salinity alters various physiological and biochemical processes in plants and thus affects their growth and development [12,13]. In the germination phase, salinity influences the percentage of germinating seeds and germination vigor [11]. Moreover, it has also been shown that all yield parameters and pigment (chlorophylls and carotenoids) content decreased under increasing salinity [11].

High salt concentration induces damage in proteins, lipids, and nucleic acid, leading to increased ROS [12]. Previous studies revealed increased hydrogen peroxide (H_2_O_2_) in wheat and sorghum, as the enhancement of H_2_O_2_ strongly depends on the tolerance of plant genotypes [11,14]. Activating the antioxidant enzymes and synthesis of protective components are very important for the survival of plants under salt stress [11,15].

Photosynthesis is among the processes strongly influenced by plant salt stress [2]. Salinity affects the structure and composition of the thylakoid membranes, which leads to the inhibition of photosynthetic activity. A previous study has shown high sensitivity of the processes in the photosynthetic apparatus under salinity [16]. The studies of three durum wheat showed a decrease in the excitation energy trapped in photosystem II (PSII) (TRo/CSm) and energy used for electron transport (ETo/CSm) as well as an increase in the energy dissipation of PSII (DIo/CSm) depending of the salt sensitivity of the genotype [11]. The authors concluded these parameters could be used as non-invasive parameters differentiating durum wheat accessions regarding salinity tolerance. It has been shown that salt stress influences gene expression, affecting the pigment composition and organization of pigment-protein complexes of the photosynthetic apparatus [11]. Previous investigations revealed salt-induced changes in the light-harvesting complex of PSII (LHCII), inner antenna of PSII, D1 protein, and oxygen-evolving complex (OEC) as well as the photosystem I (PSI) complex [17,18]. This issue has shown that all these changes influence the energy transfer has been pigment-protein complexes of PSII, and the redistribution of the excitation energy between PSII and PSI in *Paulownia* lines with different salt sensitivity [19]. In addition, the authors revealed an inhibition of the photochemical activities of PSII and PSI and an influence on the kinetic parameters of the oxygen-evolving reactions due to the differences in the modification of the Mn cluster. The study demonstrated the relationship between the salt tolerance of *Paulownia* lines and registered changes in the energy transfer between the pigment-protein complexes and alteration of the Mn cluster of the oxygen-evolving complex under salt stress [19].

## 3. Drought Stress

Drought is an environmental factor that negatively impacts plant growth and crop production. As a result of climate change, this factor is manifested more and more often, and its duration has been prolonged in recent years [20,21]. The important role in plant growth and development and the tolerance against abiotic stress, including drought, have growth regulatory factors (GRF). A recent investigation identified 16 GRF genes from the genomes of *Medicago truncatula* and *Medicago sativa* [22]. It has also been shown that the expression levels of gene pair MtGRF2-MsGRF2 and MtGRF6-MsGRF6 are involved in drought and salt tolerance.

The processes of photosynthesis are essential for plant growth, which is strongly influenced by drought. [23]. Earlier studies showed an imbalance between light and dark reactions due to the low CO_2_ uptake caused by stomatal closure [24]. The influence of the photosynthetic rate is a result of the reduction of photosynthetic pigments and damage to the components of the electron transport chain as the OEC, dissociation of the LHCII from the reaction centers of the PSII, and D1 polypeptide degradation [25]. Plants have different molecular mechanisms to decrease the negative impact of drought stress. It is crucial for the survival of plants under water deficiency, activation of the antioxidant system, and non-enzymatic antioxidants. Moreover, plants protect the photosynthetic apparatus by dissipating the excess light energy and stimulating the cyclic electron transport around PSI [26].

It has been reported that overexpression of miRNAs decreases the sensitivity to drought-influencing target gene SPL13 by accumulating the osmoprotective compounds, proline, ABA, and antioxidants [27,28]. Jing et al. [29] investigate the molecular mechanism of the rhizobium symbiosis influencing drought tolerance. The authors revealed that seedlings with active nodules exhibited enhanced drought tolerance due to the early effects of the symbiotic nitrogen fixation (SNF) triggered in contrast to the drought susceptible with inactive nodules. The study provides insight into ceRNA involvement in rhizobium symbiosis contributing to the drought tolerance and provides molecular evidence for future studies.

## 4. Temperature Stress

Very often, plants are subjected to temperature fluctuations. Several reports indicate that extreme temperature (low or high) influences plant metabolism, leading to changes in plant growth, a decrease in chlorophyll content, membrane damage, and inhibition of photosynthesis [2]. Liu et al. [30] studied the role of Thaumatin-like proteins (TLPs) in the low-temperature response in *Ammopiptanthus nanus*. The authors revealed that several AnTLP genes are involved in the cold-stress response. It has also been shown that a cold-induced AnTLP gene, AnTLP13, was localized in the apoplast, and heterologous expression of the AnTLP13 in *Escherichia coli* and yeast cells and tobacco leaves enhanced the tolerance to the low-temperature stress in cells or seedlings.

## 5. Effects of Herbicides

The extensive use of herbicides in agriculture leads to a rise in soil levels. Some herbicides used in agriculture are acetyl-CoA carboxylase inhibitors (ACCase-inhibitors). Acetyl-CoA carboxylase is involved in fatty acid synthesis, a crucial process in forming cellular membranes and plant lipids [31]. Mo et al. [31] compare the population of *Polypogon fugas* resistant to ACCase-inhibitors with others sensitive to these inhibitors. Molecular analyses showed that the resistant population presented an amino acid mutation (Asp-2078-Gly) in comparison to the ACCase gene sequences of the sensitive population.
